# Supplementary material for: The genome sequence of Filago lutescens Jord., 1846 (Asterales: Asteraceae)
Source: Wellcome Open Res. 2026 Jul 8;11:428. [Version 1] doi: 10.12688/wellcomeopenres.26896.1 (PMC13563012; doi:10.12688/wellcomeopenres.26896.1)
Supplement: Peer review history

# Reviewer report: Reviewer response for version 1

**DOI:** 10.21956/wellcomeopenres.29615.r164305

**Published:** 2026-09-10

**Authors:** Evangelos C Tatsis

**Affiliations:** 1 Hainan University, Sanya, China

This Data Note reports a Darwin Tree of Life reference genome for *Filago lutescens* (Red-tipped Cudweed), a rare species in Britain. The work follows the established DToL pipeline: PacBio HiFi (28.00 Gb, ~37×), Arima Hi-C for scaffolding, hifiasm in Hi-C phasing mode, YaHS scaffolding, manual curation in PretextView, and Ensembl annotation supported by leaf RNA-seq.

 The technical quality of the assembly is high and, on the metrics presented, clearly exceeds the EBP reference standard: consensus QV of 70.2 (hap1) and 72.1 (hap2), k-mer completeness above 99.6% for both haplotypes, contig N50 of 5.24 Mb, scaffold N50 of 43.62 Mb, and 99.77% of the sequence assigned to 14 chromosomal pseudomolecules. Methods are described in the standard DToL format with software versions, accessions, and protocols.io links, and all data are openly deposited under PRJEB74261. The note is a useful and well-executed resource, and I am supportive of it.

 My reservations are almost entirely about interpretation and completeness of reporting rather than about the data themselves. Addressing these the points below would, in my view, substantially raise the value of this note for very little additional work.

 1. The BUSCO duplication rate (D = 60.6%) is the most striking result in the paper and is not mentioned once in the text. Table 4 reports C:98.4% [S:37.8%, D:60.6%] against an EBP benchmark of S > 90% and D < 5%, i.e. the assembly fails one of the benchmarks it is being scored against by an order of magnitude, and this passes without comment. In the Results the figure is quoted neutrally as "98.4% of the expected gene set". My question is if this is biological or technical.

 2. GenomeScope2 gives a haploid size of 771.30 Mb and flow cytometry gives 730.00 Mb (1C = 0.75 pg), yet the assembly spans 648.45 Mb — a shortfall of roughly 82 Mb (11%) and 123 Mb (16%) respectively. At present the text simply states that the two estimates "guided expectations for the assembly" and moves on. Please state the discrepancy explicitly and offer the likely explanation.

 3. The note reports 14 pseudomolecules but never states what the expected chromosome number is. Filago is consistently reported as 2n = 2x = 28 (x = 14), so the assembly is in agreement with the cytological literature — this is a positive result, and it is being left on the table. A single sentence in the Background or the Genome sequence report giving the expected number with a citation, and noting the allopolyploid ancestry of the clade, would strengthen.

 4. The annotation section needs more detail and one clarification.

 5. Organelle assemblies need one or two more sentences.

 6. Collection of a statutorily protected species. The Background states that F. lutescens is rare and statutorily protected in Britain, with only 14 sites recorded since 1990. The note relies on the generic DToL Sampling Code of Practice statement. For a species of this conservation status, I would expect an explicit statement that collection was made under the appropriate license and with landowner permission, and I would also expect an herbarium voucher accession (specimen ID KDTOL10631 is given, but no herbarium barcode). Please add both.

Are sufficient details of methods and materials provided to allow replication by others?

Yes

Is the rationale for creating the dataset(s) clearly described?

Yes

Are the datasets clearly presented in a useable and accessible format?

Yes

Are the protocols appropriate and is the work technically sound?

Yes

Reviewer Expertise:

plant specialised metabolism, medicinal plant genomics

I confirm that I have read this submission and believe that I have an appropriate level of expertise to confirm that it is of an acceptable scientific standard.

# Reviewer report: Reviewer response for version 1

**DOI:** 10.21956/wellcomeopenres.29615.r164307

**Published:** 2026-09-08

**Authors:** Marco Todesco

**Affiliations:** 1 Michael Smith Laboratories, University of British Columbia, Vancouver, Canada

This data note reports the genome assembly of red-tipped cudweed ( *Filago lutescens*), as part of the Darwin Tree of Life (DToL) project. The sequencing, assembly, and annotation process followed established DToL pipelines, and resulted in a highly complete, chromosome-level assembly.

 The *F. lutescens *assembly is clearly of very high quality, and more than satisfies the EBP target summary metrics. Puzzingly, the level of duplication in BUSCO analyses is very high (>60%), despite there being little evidence for widespread artificial duplications in the assembly (the assembly size is slightly smaller than the predicted size). Nonetheless, this assembly will be a useful resource for comparative genomics analyses within the Asteraceae family.

Are sufficient details of methods and materials provided to allow replication by others?

Yes

Is the rationale for creating the dataset(s) clearly described?

Yes

Are the datasets clearly presented in a useable and accessible format?

Yes

Are the protocols appropriate and is the work technically sound?

Yes

Reviewer Expertise:

plant genomics, plant genetics, adaptation

I confirm that I have read this submission and believe that I have an appropriate level of expertise to confirm that it is of an acceptable scientific standard.

# Reviewer report: Reviewer response for version 1

**DOI:** 10.21956/wellcomeopenres.29615.r164311

**Published:** 2026-08-18

**Authors:** Ruslan Kalendar

**Affiliations:** 1 University of Helsinki, Helsinki, Finland

This Data Note reports a Darwin Tree of Life (DToL) reference genome for Filago lutescens (Red-tipped Cudweed), a nationally scarce annual of open, disturbed ground in Britain. The assembly was produced from a single Surrey specimen (ToLID daFilLute1) using PacBio HiFi (28.00 Gb, 37×), Arima Hi-C scaffolding and Illumina RNA-seq, following the standard WSI Tree of Life pipeline. Haplotype 1 (648.45 Mb) is curated to chromosome level with 99.77% of the sequence in 14 pseudomolecules; haplotype 2 (646.22 Mb) is left at scaffold level. Organelle genomes and an Ensembl annotation (38,942 protein-coding genes) are also reported.

 The work is competently executed, the pipeline is well established and appropriately cited, and all raw data and assemblies are deposited in INSDC under PRJEB74261. The genome is a valuable resource: F. lutescens is a rare arable-margin specialist of conservation concern, and there are very few chromosome-level references in Gnaphalieae. I recommend indexation, but a small number of substantive points should be addressed first chiefly the very high BUSCO duplication, which the manuscript does not mention at all, and an apparent inconsistency in the reported collection locality.

 1. GenomeScope reports heterozygosity of 0.01% (ab = 0.00776% in the Figure 2 header), and the MerquryFK plot (Figure 4) shows the hap1-only and hap2-only curves lying essentially flat on zero i.e. there are almost no haplotype-specific k-mers. The two assemblies are therefore not two meaningfully distinct haplotypes but two near-identical copies, consistent with a highly selfing or strongly inbred annual. The manuscript reports the numbers but never draws the conclusion. Please state this explicitly so that users of the data do not treat hap2 as an independent haplotype for, e.g., allele-specific analyses.

 2. The assembly span (648.45 Mb) was 11% below the flow cytometric 1C estimate (730 Mb) and 16% below the GenomeScope estimate (771.30 Mb). With 42.31% non-unique k-mer content and only 37× HiFi coverage, the collapse of high-copy repeats (satellite arrays, rDNA) is an obvious candidate, but the manuscript does not comment on this.

Are sufficient details of methods and materials provided to allow replication by others?

Yes

Is the rationale for creating the dataset(s) clearly described?

Yes

Are the datasets clearly presented in a useable and accessible format?

Yes

Are the protocols appropriate and is the work technically sound?

Yes

Reviewer Expertise:

bioinformatics, comparative genomics

I confirm that I have read this submission and believe that I have an appropriate level of expertise to confirm that it is of an acceptable scientific standard.
